# HIV Self-Testing in Lusaka Province, Zambia: Acceptability, Comprehension of Testing Instructions, and Individual Preferences for Self-Test Kit Distribution in a Population-Based Sample of Adolescents and Adults

**DOI:** 10.1089/aid.2017.0156

**Published:** 2018-03-01

**Authors:** Arianna Zanolini, Jenala Chipungu, Michael J. Vinikoor, Samuel Bosomprah, Mazuba Mafwenko, Charles B. Holmes, Harsha Thirumurthy

**Affiliations:** ^1^ Centre for Infectious Disease Research in Zambia, Lusaka, Zambia.; ^2^ American Institutes for Research, Lusaka, Zambia.; ^3^ School of Medicine, University of Alabama at Birmingham, Birmingham, Alabama.; ^4^ School of Medicine, University of Zambia, Lusaka, Zambia.; ^5^ School of Medicine, Johns Hopkins University, Baltimore, Maryland.; ^6^ Gillings School of Global Public Health, University of North Carolina at Chapel Hill, Chapel Hill, North Carolina.

**Keywords:** HIV/AIDS, HIV self-testing, HIV prevention, discrete choice experiment, Africa, population-based survey

## Abstract

We assessed attitudes and preferences toward HIV self-testing (HIVST) among Zambian adolescents and adults. We conducted a population-based survey of individuals aged 16–49 years old in Lusaka Province, Zambia. HIVST was shown to participants through a short video on oral fluid-based self-testing. In addition to demographics, HIV risk perceptions, and HIV testing history, we assessed participants' acceptability and concerns regarding HIVST. Using a discrete choice experiment, we investigated preferences for the location of self-test pickup, availability of counseling, and cost. After reviewing an instructional sheet or an additional video, we assessed participants' understanding of self-test performance. Among 1617 participants, 647 (40.0%) were male, 269 (16.6%) were adolescents and 754 (46.6%) were nontesters (i.e., no HIV test in the past 12 months). After viewing the video, 1392 (86.0%) reported that HIVST would make them more likely to test and while 35.0% reported some concerns with HIVST, only 2% had serious concerns. Participants strongly preferred HIVST over finger prick testing as well as having counseling and reported willingness to pay out-of-pocket (US$3.5 for testers and US$5.5 for nontesters). Viewing an HIVST demonstration video did not improve participant understanding of self-test usage procedures compared to an instructional sheet alone, but it increased confidence in the ability to self-test. In conclusion, HIVST was highly acceptable and desirable, especially among those not accessing existing HIV testing services. Participants expressed a strong preference for counseling and a willingness to pay for test kits. These data can guide piloting and scaling-up of HIVST in Zambia and elsewhere in Africa.

## Introduction

Low uptake of HIV testing services in sub-Saharan Africa (SSA) is among the main barriers to achieving the 90-90-90 targets established by UNAIDS^1^ and to realizing the promise of HIV treatment as prevention.^2^ About 50% of HIV-positive persons in SSA are unaware of their HIV status, and awareness is particularly low among men.^1^ While approaches that bring testing services into communities may overcome standard barriers to clinic-based testing,^3,4^ there remains a need for additional testing approaches that can be accessed by otherwise hard-to-reach segments of the population and that can facilitate more frequent testing.^4,5^

HIV self-testing (HIVST) is a promising approach that can address multiple barriers associated with HIV testing.^6–8^ Self-tests, which enable individuals to test themselves for HIV privately and at their own convenience, can circumvent a number of structural, psychosocial, and health systems barriers to HIV testing. A growing number of studies have begun to show that HIVST is highly acceptable in various populations and settings.^9–11^ Simple oral fluid-based tests have achieved high sensitivity and specificity, prompting several countries in SSA to develop policies for implementation and support of HIVST.^12^ Several countries have begun to explore ways to make self-tests more widely available and the World Health Organization is preparing HIVST guidelines.^13^ However, country-specific evidence on acceptability as well as optimal pricing and distribution strategies for HIVST remain lacking. In particular, data are needed to inform decisions regarding ideal delivery strategies and the implications of alternative packaging and pricing on self-test uptake.

In Zambia, where HIV testing coverage remains below the UNAIDS' target, the Ministry of Health (MoH) has called for investigation of HIVST as a new approach to improve uptake of HIV testing.^14,15^ Understanding HIVST preferences of individuals who are less likely to have tested for HIV in the past is particularly important to maximize the impact of HIVST scale-up. Given the challenge of conducting controlled trials to evaluate the effects of alternative prices and other “attributes” of HIVST, discrete choice experiments (DCE) may be useful for informing policy decisions about such new technologies. DCEs have previously been used to assess preferences for various new technologies or services.^16,17^ To guide HIVST policy in Zambia and the region we conducted a population-based survey of HIVST acceptability among adolescents and adults and utilized a DCE to understand preferences regarding HIVST delivery.

## Materials and Methods

### Study design and participants

The study was conducted in urban and rural areas of Zambia's largest province, Lusaka Province, which has a population of 2.1 million and adult HIV prevalence of 16.3%.^18^ A two-stage sampling strategy was used to select survey participants. First, we selected 13 Census Supervisory Areas (CSAs) in Lusaka urban district and 4 CSAs in rural districts using probability proportional to size sampling. We used the 2010 Zambian Census to inform our sampling strategy and to define the boundaries of each district. In each selected CSA, research assistants approached 100 households using either the linear or the spiral approach (approaches used routinely in the census) depending on the geographical distribution of households and approached every third house starting from a place in a randomly selected subdivision of the CSA. Within each household, research assistants selected one person aged 16–49 years using a computer-generated list of random numbers. In the event that the selected person was not at home, a maximum of three repeat visits were attempted before dropping the observation to obtain complete data. Written informed consent was obtained from each participant.

### Data collection procedures

We used a structured survey questionnaire to measure participant demographic characteristics, HIV testing history, knowledge of available HIV services, self-reported sexual behavior, and HIV risk perceptions. The survey was administered in the preferred languages of participants (Nyanja, Bemba, Tonga, or English) and data were collected on tablet computers that were equipped with a SIM card and data plan to transmit data back to the central server. Research assistants carried solar chargers to maintain tablet power in field settings.

HIVST was not available in Zambia at the time of data collection. Therefore, research assistants first showed participants a professionally developed video to introduce the concept of HIVST. Use of the video allowed us to standardize the information provided to participants and limit interviewer-specific discrepancies in the content and quality of information provided. In the video an oral fluid-based HIV test was demonstrated (OraQuick Advance HIV-I/2; OraSure Technologies, Bethlehem, PA). After watching the video, participants were administered a series of questions to assess their demand for self-tests and their concerns about HIVST.

Research assistants also administered a DCE, a methodology used to elicit individuals' preferences. In a typical DCE conducted in healthcare settings, participants choose between two or more products, services, or models of healthcare, some of which may be hypothetical ones. The assumptions behind DCE are that the participant's choice of a model reveals his or her preference, and that preference for a model can be broken up into preferences for the attribues that define that model. Each model is defined by a few attributes that can take different levels in each model. As participants choose between models with varying attributes, they reveal their preference for each of these attributes.

In our study, the DCE consisted of nine questions and in each question participants were given three models, one of which was considered the standard, and asked to chose the one that would would make them more likely to test for HIV in the next 6 months. Those who reported taking an HIV test in the past year were given two HIVST options and a standard option that included fingerprick HIV testing in a facility (i.e., HIV testing standard of care in Zambia). Nonregular testers (i.e., no HIV test in past year) were given two HIVST models with the third option being not taking an HIV test. After piloting, we decided to use pictures to represent attributes. An example question is shown (Supplementary Fig. S1; Supplementary Data are available online at www.liebertpub.com/aid). Based on discussions with the Zambian MoH, HIV testing experts, and community representatives, we included the following attributes in the DCE: location to obtain a self-test [at voluntary counseling and testing (VCT) located in a health facility, at a health facility's outpatient pharmacy, or at a community private pharmacy], cost [free, 1.5 U.S. dollar (USD), or 3.5 USD], and whether counseling was available (yes or no; Supplementary Fig. S1). Using a D-efficient design^19,20^ we created a blocked design with two versions (one for “testers” and one for “non-testers”) each containing nine questions.

Finally, participants were asked questions that examined their comprehension of how to correctly use self-tests after being shown intructions in two ways. First, participants were given ∼1 min to review an instructional sheet with visual aids adapted from one originally developed for the Thailand market and previously used in research in Kenya.^21^ Then, at random, half the participants were additionally allowed to view a 1-min video produced for the study that featured a woman demonstrating how to use an oral fluid-based HIV test (Oraquick HIV-1/2 antibody test; OraSure Technologies). All participants were subsequently asked how confident they were that they understood how to do the test. A 9-item questionnaire was used to objectively assess their understanding. Participants in both groups were allowed to review the instructional sheet when responding.

### Statistical analyses

We described participants' responses through means and standard deviations, and we also compared means across different strata to tests whether certain sub-groups had distinct HIVST preferences. Acceptability was defined as responding “very comfortable” or “comfortable” in three questions asking about level of comfort with using a self-test, in giving it to friends, and in giving a self-test to someone whom they know. Understanding of the instructions was measured as mean and standard deviation in a 9-item comprehension test with open and multiple choices (Supplementary Table S1). Using probability weighting, we weighted data to ensure representativeness. We described acceptability by summarizing participants' responses to questions about their own comfort level with and likelihood of using a self-test, as well as their perceptions about the comfort level of others with HIVST.

Participants' preferences for HIVST and the relative weights given to various attributes of HIVST were examined by analyzing responses to the DCE. We analyzed DCE data using mixed logit regression with a random coefficient model and allowed the attribute of counseling to vary between participants. We verified robustness of results using conditional logits. Estimated coefficients were interpreted as the marginal change in utility from altering the attribute. Larger coefficients for an attribute implied stronger preferences for that attribute, with other factors being constant. Cost was included as a categorical variable, but also analyzed as continuous when calculating willingness to pay, in line with previous DCE literature. We calculated willingness to pay for self-tests as the inverse of the ratio between the coefficient for presence of self-testing in the model and cost. The mixed logit model was chosen because it allows for heterogeneity of preferences and is advisable when questions are clustered at the participant level. It also does not make the assumption of independence of irrelevant alternatives. The percentage of participants with a positive preference for HIVST and counseling was calculated using the value of the cumulative standard normal for the ratio between the coefficient and the standard deviation of the coefficient. During data collection, 1 USD was equal to 7 Zambian Kwacha (ZMW).

Finally, participants' understanding of how to use self-tests was interpreted by calculating the proportion who felt comfortable using self-tests and summing the responses to the nine knowledge assessment questions. Mean scores were compared for the two groups that received alternative testing instructions using a *t*-test. All data were analyzed using Stata, version 14 (Statacorp, College Station, TX). The study received ethical approval from the Excellence in Research Ethics and Science Converge (Lusaka, Zambia).

## Results

Among 1912 houses randomly selected for the survey in Lusaka Province, 1617 adolescents and adults participated (representing 84.6% of households). During recruitment, 229 selected houses were empty, 49 had an adult who could not be reached, and 17 had an adult who declined participation. We interviewed an average of 95 participants per CSA and 77% of the participants resided in urban areas.

Among participants, 970 (60%) were women and 647 (40%) were men (Table 1). Participants' median age was 27 years (interquartile range, 22–35); 50% were married and 47% had completed senior secondary school or above. Moderate or high risk of HIV infection was reported by 41% and 12% self-reported being HIV-positive. Although 85% reported having ever tested, only 52% of women and 38% of men reported having tested in the past 12 months. Only 4% reported having heard of HIVST before the study.

**Table 1.** Demographic, Socioeconomic, and HIV Risk Characteristics of Participants

	*Total*		*Female*		*Men*	
	N	*Mean (SD)/%*	N	*Mean (SD)/%*	N	*Mean (SD)/%*
Age, in years	1617	28.9 (8.3)	970	28.5 (8.1)	647	29.5 (8.4)
Education level						
None/primary	1616	20	970	24	646	16
Jr. secondary		32		11		29
Sr. secondary		33		34		34
More than secondary		14		10		21
Marital status						
Married/cohabiting	1614	50	969	53	645	43
Single		38		32		47
Divorced/separated/widowed		12		14		9
Employment status						
Employed for wages	1617	23	970	14	647	37
Self-employed with business/farm		28		28		29
Unemployed, looking for work		23		27		16
Unemployed, not looking for work		26		32		17
Individual monthly income level^a^						
K500 or less	805	21	384	28	421	14
K501–1000		28		33		23
K1001–2000		21		18		25
K2001–5000		23		16		3
>K5000		7		5		8
Number of children	1599	1.8 (1.8)	970	2.0 (1.8)	647	1.5 (1.8)
HIV-positive by self-report	1325	12	846	12	479	11
Perceived HIV risk						
High	1363	14	804	15	559	13
Moderate		27		27		28
Low		31		30		32
No risk at all		28		28		27

All data were weighted using probability weighting to ensure representativeness.

^a^ At the time of the survey K7 was equivalent to 1 US dollar.

SD, standard deviation.

After learning about HIVST through the video, acceptability of HIVST was almost universal: 91% of participants reported they would be comfortable or very comfortable with using a self-test and they also felt friends (76%) and partners (86%) would feel similarly comfortable. Acceptability was high across different strata of education, income, HIV risk, and rural status (Table 2). Eighty-seven percent of participants felt that HIVST would increase their likelihood of testing. Similar results were found in analyses stratified by sex, age, and education level. The majority (85%) of participants also felt their friends would be more likely to test for HIV if self-testing was available. Notably, 76% of those who had not tested recently declared they were “very sure” they would use a self-test if it was available. A minority (35.0%) reported having any concerns about HIVST and multiple different concerns were reported. Concern regarding possible suicide (reported by 8.1%) and lack of counseling after self-testing (reported by 6.3%) were the two most common concerns reported by participants. Most concerns were not rated as major concerns and only 2.1% reported that their concerns should limit the availability of HIVST in Zambia (Table 3).

**Table 2.** Acceptability of HIV Self-Testing in Population-Based Sample of Zambian Adolescents and Adults

	*% responding “comfortable” or “very comfortable” with HIVST in these scenarios*				*Reported likelihood of HIV testing with HIVST*		
	*Self use*^a^	*Friend use*^b^	*Give to partner*^c^	*Give to friend or other*^d^	*% more likely to take HIV test*	*% friends more likely to HIV test*	*% would take HIVST “for sure”*
No. of respondents	1605	1439	1430	1589	1600	1386	626
Overall, %	91	76	86	79	87	85	76
Sex, %							
Female	91	77	87	82	88	86	79
Male	90	73	85	76	86	83	73
HIV testing history, %							
Nontester	91	73	84	79	86	85	76
Tester	91	77	88	80	88	85	N/A
Education level, %							
Lower	90	74	84	79	85	83	74
Higher	92	77	89	80	89	87	79
Income level, %							
Lower	91	75	85	78	85	82	74
Higher	91	78	89	78	85	87	79
Perceived HIV risk, %							
Higher risk	93	77	87	76	90	84	76
Lower risk	91	75	87	80	88	85	80
Location, %							
Rural	94	75	88	81	87	86	71
Urban	90	76	86	79	87	85	77

^a^ How comfortable would you feel using a self-test?

^b^ How comfortable would your friends feel using a self-test?

^c^ How comfortable would your partner feel if you have it to him/her?

^d^ How comfortable would you feel giving it to your friends or acquaintances?

HIVST, HIV self-testing.

**Table 3.** Concerns of Adolescents and Adults Regarding HIV Self-Testing in Zambia

	%	*% of overall sample*
% with no concerns regarding HIVST		65.0
% with any concern regarding HIVST		35.0
Types of concerns^a^		
Suicide	23	8.1
Lack of postcounseling and mental health	18	6.3
Lack of linkage to care	12	4.2
Validity of the test results	12	4.2
Lack of behavioral postcounseling advice	11	3.9
Intimate partner violence	8	2.8
Other	8	2.8
Coercion of people to take an HIV test	5	1.8
High cost	3	1.1
Severity of the concern^b^		
Very severe and should limit HIVST	6	2.1
Important but can be addressed	71	24.9
Relatively minor	24	8.4

^a^ Participants were allowed to list more than one concern.

^b^ Severity was elicited only for the first concern mentioned by each participant.

In the DCE, for both testers and nontesters, self-testing (vs. the standard) and having counseling were the main drivers of their choice to get tested (Table 4). For nontesters, presence of self-testing was slightly more important than presence of counseling when determining their choice, while the opposite was true for testers. The strength of the preference for self-testing was higher for nontesters than for testers (2.91 over 1.65). Both testers and nontesters valued counseling, and an estimated 81% of nontesters and 83% of testers had a positive preference for counseling. Compared to self-testing and counseling, preference for location was modest, with participants expressing a nonsigificant preference for picking up a self-test at the outpatient department versus VCT. Cost had a negative and significant coefficient, indicating that participants preferred lower cost self-tests. Testers were more sensitive to cost than nontesters. Willingness to pay for self-tests was 3.3 USD for testers and 4.6 USD for nontesters. Willingness to pay was higher as income and education levels increased and for urban compared to rural participants. When conditional logit was used in secondary analyses, preferences for counseling, location, and cost remained qualitatively similar and when we excluded questions where participants chose the status quo and only considered the choice between the two self-testing models.

**Table 4.** Patient Preferences Regarding HIV Self-Testing According to a Discrete Choice Experiment

	*Regular HIV testers*		*Nonregular HIV testers*	
*Attributes*	*Coefficient*^a^	*SE*	*Coefficient*^a^	*SE*
Self-testing (vs. status quo^b^)	1.65	0.05	2.91	0.07
Counseling (vs. no counseling)	2.75	0.12	2.55	0.14
Location of self-test pickup				
OPD pharmacy at clinic	0.03	0.05	0.10	0.06
Chemist	−0.16	0.05	−0.06	0.06
VCT office at the clinic	Reference	N/A	Reference	N/A
Cost of self-test kit	−0.07	0.00	−0.09	0.00

^a^ A random coefficient logistic regression model was used. Separate models were performed for participants who regularly took HIV tests under the status quo and those who had not tested. All attributes were adjusted for in models.

^b^ The status quo was finger prick point-of-care HIV testing performed in the facility VCT office for regular testers and not testing for nonregular testers.

OPD, outpatient department; SE, standard error; VCT, voluntary counseling and testing.

The instructional sheet for oral fluid-based self-tests performed well with 73% of participants reporting they felt confident in their ability to use the test themselves (Table 5). Participants with lower socioeconomic status were less likely (53%) to feel confident about using the test. Responses to the knowledge assessment questions also indicated good understanding of HIVST (median score: 8 out of 9 correct, range 2–9). Most incorrect answers were to the question related to interpretation of test results (65% accurately identified the image of invalid, positive, and negative test results). Those with lower education levels were less likely to correctly interpret test results compared to those with high education levels (44% vs. 70%; *p* < .01). Supplementing the instruction sheet with a brief video increased participant confidence slightly (79%) but did not improve (and slightly worsened) understanding of how to perform the test.

**Table 5.** Effectiveness of HIV Self-Testing Instructions

	*Paper instructions only*			*Paper plus video instructions*			
	*Overall*	*Higher education*^a^	*Lower education*	*Overall*	*Higher education*^a^	*Lower education*	p^b^
Confidence in performing an HIV Self-test							
% not confident	5	3	7	5	3	5	.01
% somehow confident	22	14	28	17	11	22	
% confident	73	83	64	79	87	72	
HIVST knowledge quiz							
% with correct number of minutes for valid test	94	96	91	90	95	80	<.01
% with correct response on reading results	65	70	44	61	67	35	.02
Mean number of correct answers out of 9 (SD)	8.1 (1.2)	8.3 (1.0)	7.9 (1.3)	7.9 (1.3)	8.2 (1.1)	7.6 (1.4)	<.01
% incorrectly said it was okay to drink water	73	72	73	71	67	77	.51

^a^ Higher education was defined as completion of secondary school and above.

^b^ *p*-Value refers to comparison of proportions between participants who received paper only versus paper and video instructions.

## Discussion

In a representative survey of adolescents and adults living in Lusaka Province, Zambia, HIVST was found to be highly acceptable and participants expressed relatively few concerns regarding the introduction of HIVST. Importantly, those who had not recently tested reported strong willingness to learn their HIV status through a self-test. The use of tablets was both highly feasible and facilitated communication around HIVST even though actual self-tests were not available in Zambia at the time of the survey. Participants also expressed a strong preference for counseling to be made available with HIVST as well as a willingness to pay US$3–5 out-of-pocket for test kits. Together these data can guide piloting and scaling-up of HIVST in Zambia and the region. Strengths of the study include its relatively large sample size and representativeness of the largest Province in Zambia, which enhances external validity of the results. Participant characteristics were similar to those from the recent Zambia Demographic and Health survey.^22^ By utilizing a DCE, the study also enhances understanding of individuals' preferences for HIVST without having provided self-tests.

Consistent with formative studies in other SSA countries,^8,21,23^ HIVST was highly acceptable. Compared to those who had tested in the past year, those who had not expressed stronger interest and preference for HIVST, including a higher willingness to pay for self-tests, suggesting that HIVST can be useful in efforts to meet the first of the UNAIDS 90-90-90 targets. Beyond the strong interest in own use of self-tests, participants also felt their friends would have strong interest in self-testing. Although the risk of suicide after receipt of positive self-test result and general lack of psychological support after testing were the most common concerns among study participants, a very small proportion of all participants expressed such concerns. Despite the stated concerns, it is notable that no studies of HIVST implementation to date have reported instances of suicide resulting from self-test use.^6,21,24^

The DCE results provided important new insights for the scale-up of HIVST. Zambian adults expressed preference for HIVST over current HIV testing approaches and on average expressed a strong preference that counseling should accompany HIVST. This may reflect very low knowledge about this new testing approach in Zambia or suggest that people strongly value psychological counseling provided by health workers. HIVST scale-up should therefore be accompanied by adequate information about how and where to access confirmatory testing services in the event of a positive result, as well as innovative ways to access pre- and post-test counseling though services such as phone-based hotlines. Further exploration of how counseling can be provided is necessary.

Another important result is that cost did not appear to be a major barrier to self-test use. Data from our study suggest willingness to pay about US$3 per self-test, with those who had not tested recently being willing to pay an additional US$2. One limitation here is that willingness to pay estimates are average amounts based on a simple model with considerable residual variation remaining from specifying the utility only on attributes of location, cost, and counseling. However, it is possible that willingness to pay for self-tests is high in light of transport costs, opportunity costs, and stigma associated with seeking HIV testing at health facilities. These results also have implications for how best to target self-tests to those who are least likely to use existing HIV testing services. Further dedicated research on pricing is needed, including studies that estimate prices using field experiments in which price is randomly assigned to potential buyers and buying choice at each price is observed.^25,26^

Most participants were able to correctly understand self-test usage procedures after reviewing an instructional sheet, and the addition of a video demonstration did not further improve understanding though it did increase confidence about using a self-test. Videos about self-test usage may be a valuable way to increase demand in settings like Zambia. While our study did not include use of actual self-tests, the accuracy with which test results were interpreted was not high. This may be the result of the survey setting and a short introduction to HIVST, but it does suggest the need for adequate supportive materials and resources when distributing self-tests. Notably, in Malawi a study that included a brief demonstration before provision of self-tests indicates high accuracy in the hands of lay users.^24^

Several limitations warrant discussion. Although both urban and rural individuals participated in the survey, our data may not generalize to all of Zambia as we only included one province of Zambia. A second limitation is that we did not recruit key populations who have both higher risk of HIV infection and may have lower access to testing in current programs. While studies show high acceptability of HIVST among key populations,^7^ additional research is needed as these populations may have different preferences around how to obtain self-tests and willingness to pay for self-tests. Similarly, very affluent individuals (a group not oversampled in this survey) in settings like Zambia may have unique perspectives and preferences for HIVST. Finally, this study focused on oral fluid based self-tests. Further studies are needed to also understand preferences for blood-based self-tests.

In Lusaka Province, Zambia, a representative group of adolescents and adults reported a high level of interest and acceptance of HIVST, particularly among those who do not regularly access HIV testing through current programs. Participants reported strong preference for presence of counseling, a relatively weak preference for location, and a willingness to pay out of pocket for test kits, higher for nonregular testers. These results support pilot introduction of HIVST in Zambia. However, future research is needed in this area to best guide governments on the most efficient and effective use of this public health intervention.

## Supplementary Material

Supplemental data

Supplemental data
