# Supplementary material for: HIV Self-Testing in Lusaka Province, Zambia: Acceptability, Comprehension of Testing Instructions, and Individual Preferences for Self-Test Kit Distribution in a Population-Based Sample of Adolescents and Adults
Source: AIDS Res Hum Retroviruses. 2018 Mar 1;34(3):254–60. doi: 10.1089/aid.2017.0156 (PMC5863088; doi:10.1089/aid.2017.0156)
Supplement: Supplemental data [file Supp_Figure1.pdf]

Supplementary Data

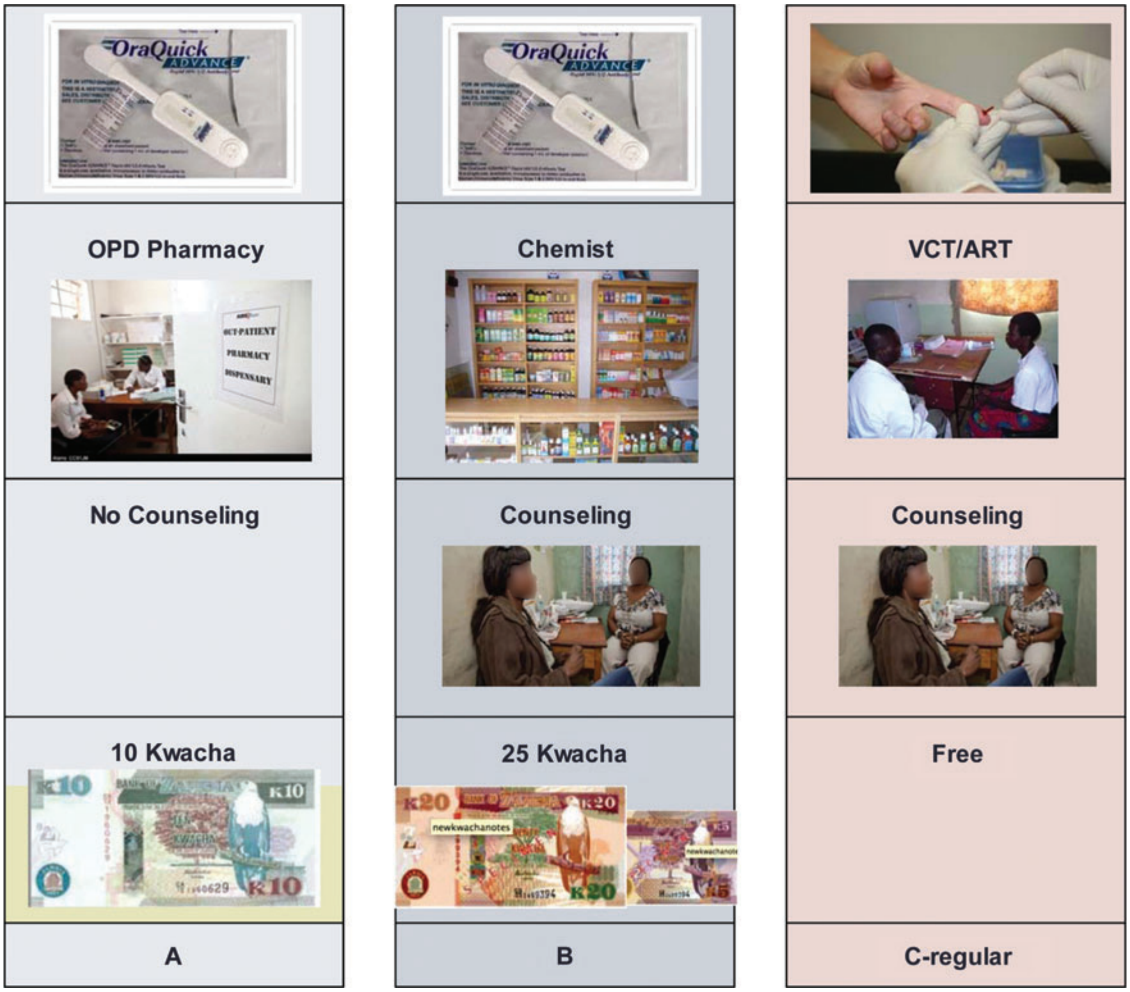

SUPPLEMENTARY FIG. S1. Example question from the discrete choice experiment.
